# *N*-acetyltransferase 2 and bladder cancer: an overview and consideration of the evidence for gene–environment interaction

**DOI:** 10.1054/bjoc.2000.1265

**Published:** 2000-07-03

**Authors:** J Green, E Banks, A Berrington, S Darby, H Deo, R Newton

**Affiliations:** Imperial Cancer Research Fund Cancer Epidemiology Unit, University of Oxford, Gibson Building, Radcliffe Infirmary, Oxford OX2 6HE, UK

**Keywords:** bladder cancer, NAT2, genetic polymorphism, smoking, arylamines, gene–environment interaction

## Abstract

Genetic polymorphism of the carcinogen metabolizing enzyme *N* -acetyl transferase 2 (NAT2) may influence susceptibility to bladder cancers related to smoking or to occupational exposure to arylamine carcinogens. This article reviews the results of 21 published case–control studies of NAT2 polymorphism and bladder-cancer risk, with a total of 2700 cases and 3426 controls. The published evidence suggests that NAT2 slow acetylator phenotype or genotype may be associated with a small increase in bladder cancer risk. However, given the possibility of selective publication of results from studies that found an excess risk, the current evidence is not sufficient to conclude that there is a real increase in risk. Only five of the 21 studies reported results separately for the effect of NAT2 on bladder cancer risk in smokers and non-smokers. Although the results suggest that the effect may be greater in smokers than in non-smokers, the possibility of publication bias makes these results difficult to interpret. There was insufficient evidence to assess the joint effect of NAT2 and occupational exposure to arylamines on bladder cancer risk. Even if estimates of the effect of NAT2 from published data are correct, studies with around 3000–5000 cases will be needed to confirm them. © 2000 Cancer Research Campaign


*British Journal of Cancer* (2000) **83**(3), 412–417
© 2000 Cancer Research Campaign
doi: 10.1054/ bjoc.2000.1265, available online at http://www.idealibrary.com on IDEAL®

### *N*-acetyltransferase 2 and bladder cancer: an overview and consideration of the evidence for gene–environment interaction

**J Green, E Banks, A Berrington, S Darby, H Deo and R Newton**

Imperial Cancer Research Fund Cancer Epidemiology Unit, University of Oxford, Gibson Building, Radcliffe Infirmary, Oxford OX2 6HE, UK

**Summary** Genetic polymorphism of the carcinogen metabolizing enzyme *N*-acetyl transferase 2 (NAT2) may influence susceptibility to bladder cancers related to smoking or to occupational exposure to arylamine carcinogens. This article reviews the results of 21 published case–control studies of NAT2 polymorphism and bladder-cancer risk, with a total of 2700 cases and 3426 controls. The published evidence suggests that NAT2 slow acetylator phenotype or genotype may be associated with a small increase in bladder cancer risk. However, given the possibility of selective publication of results from studies that found an excess risk, the current evidence is not sufficient to conclude that there is a real increase in risk. Only five of the 21 studies reported results separately for the effect of NAT2 on bladder cancer risk in smokers and non-smokers. Although the results suggest that the effect may be greater in smokers than in non-smokers, the possibility of publication bias makes these results difficult to interpret. There was insufficient evidence to assess the joint effect of NAT2 and occupational exposure to arylamines on bladder cancer risk. Even if estimates of the effect of NAT2 from published data are correct, studies with around 3000–5000 cases will be needed to confirm them. © 2000 Cancer Research Campaign

**Keywords**: bladder cancer; NAT2; genetic polymorphism; smoking; arylamines; gene–environment interaction

Genetically based variability (polymorphism) in the activity of carcinogen metabolizing enzymes may influence susceptibility to environmental carcinogens (Vineis et al, 1999). The major risk factors for bladder cancer are cigarette smoking, which is associated with a 2 to 8-fold increase in risk and may account for 50–60% of cases, and occupational exposure to arylamines such as 2-naphthylamine, 4-aminobiphenyl and benzidine, associated with an increased risk of up to 160-fold and a factor in perhaps 10–20% of cases (Hein, 1988; Silverman et al, 1996; Grant et al, 1997). Cigarette smoke contains arylamines, including 2-naphthylamine and 4-aminobiphenyl, which could explain its effects in inducing bladder cancer (Vineis, 1992; Peluso et al, 1998; Sorlie et al, 1998). There is no evidence for an appreciable role of high-penetrance genes in determining bladder-cancer risk (Cartwright, 1979; Kantor et al, 1985; Kiemeny et al, 1997).

The carcinogen-metabolizing enzyme *N*-acetyltransferase 2 (NAT2) is important in the inactivation of arylamines. There are two distinct phenotypes for NAT2: ‘fast’ and ‘slow’ acetylators, measured in vivo using substrates such as isoniazid, dapsone and caffeine. The slow form of NAT2 is present in up to 90% of some Arab populations, in 40–60% of Caucasians, including Indians, and in 5–25% of East Asians (Lin et al, 1994; Woolhouse et al, 1997; Xie et al, 1997). It has been suggested that slow acteylators may be at increased risk of bladder cancer when exposed to environmental arylamine carcinogens, due to their slower inactivation. Some but not all published case-control studies have provided support for this hypothesis (d’Errico et al, 1996; 1999).

*Received 21 February 2000*
*Revised 10 April 2000*
*Accepted 11 April 2000*

*Correspondence to:* J Green

The aims of this paper are first, to assess the evidence from published case-control studies for an association between the NAT2 slow phenotype or genotype and bladder cancer risk; and secondly, to consider whether existing evidence supports the hypothesis that the effect of the NAT2 polymorphism on bladder cancer risk will be seen primarily in those exposed to tobacco smoke or to occupational arylamine carcinogens.

### METHODS

Case-control studies of the NAT2 polymorphism and bladder cancer published up to the end of 1998 were identified through searches of Medline and Embase databases, supplemented by searches of references given in identified papers and by hand searches of relevant journals. No restriction was placed on the language of publication. No attempt was made to identify unpublished studies or unpublished data from published studies. Analyses were restricted to studies with at least 50 cases and with adequate information on exposure and outcome measures.

Unadjusted odds ratios (with 95% confidence intervals) for bladder cancer in NAT2 slow acetylators compared with NAT2 fast acetylators were calculated for each study using published figures. NAT2 slow and fast acetylators were as defined in each study according to the published methods for phenotyping or genotyping. Odds ratios were compared between groups of studies based on type of control group, method used to determine acetylation status (phenotype or genotype), and ethnic group (Caucasian (Europe, USA, India) or East Asian (Japan, Korea, Taiwan), based on ethnic origin of subjects, where given, or country where study was performed). In studies with sufficient information, odds ratios were calculated separately for smokers and for non-smokers smokers and for subjects occupationally exposed to arylamines

| Study | | Cases/Controls NAT2 slow | Cases/Controls NAT2 fast | OR |
|---|---|---|---|---|
| Brockmoller et al 1996* | *Germany* | 233/215 | 141/158 | 1.21 |
| Okkels et al 1997* | *Denmark* | 154/135 | 100/107 | 1.22 |
| Taylor et al 1998* | *USA* | 121/109 | 109/94 | 0.96 |
| Mommsen et al 1985 | *Denmark* | 145/54 | 83/46 | 1.49 |
| Risch et al 1985* | *UK* | 127/26 | 62/33 | 2.60 |
| Ladero et al 1985 | *Spain* | 83/90 | 47/67 | 1.31 |
| Lower et al 1979 | *Denmark* | 80/79 | 35/39 | 1.13 |
| Peluso et al 1998* | *Italy* | 76/26 | 38/20 | 1.54 |
| Cartwright et al 1982 | *UK* | 74/54 | 37/41 | 1.52 |
| Hanssen et al 1985 | *Germany* | 65/18 | 40/24 | 2.17 |
| Roots et al 1989 | *Germany* | 67/149 | 35/143 | 1.84 |
| Evans et al 1983 | *UK* | 66/510 | 34/342 | 1.30 |
| Kaisary et al 1987 | *UK* | 59/54 | 39/56 | 1.57 |
| Lee et al 1994 | *Korea* | 16/14 | 82/70 | 0.98 |
| Dewan et al 1995 | *India* | 46/28 | 31/52 | 2.76 |
| Ishizu et al 1995 | *Japan* | 20/13 | 51/78 | 2.35 |
| Lower et al 1979 | *Sweden* | 46/38 | 25/36 | 1.74 |
| Hanke et al 1990 | *Poland* | 47/10 | 20/12 | 2.82 |
| Sone 1986 | *Japan* | 5/11 | 57/71 | 0.57 |
| Su et al 1998* | *Taiwan* | 12/13 | 41/88 | 1.98 |
| Horai et al 1989 | *Japan* | 3/13 | 48/190 | 0.91 |
| **ALL STUDIES** | | 1545/1659 | 1155/1767 | 1.41 |

Test for heterogeneity between all studies: $\chi^2_{20}$=25.90; *p*=0.17

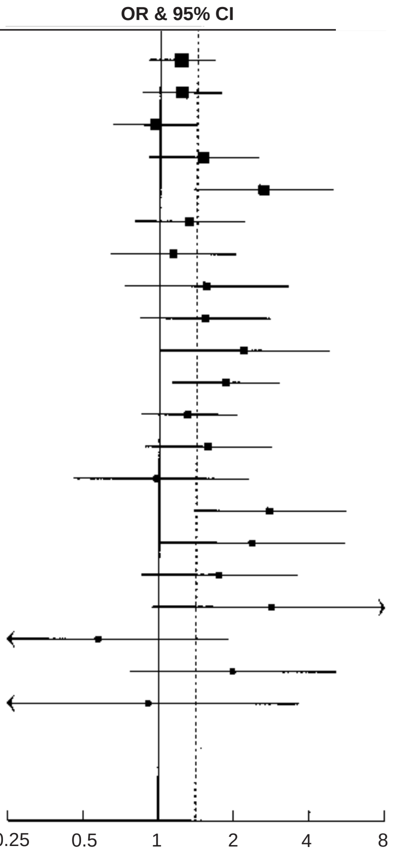

Dashed line represents point estimate of summary odds ratio for all 21 studies
* Studies involving measurement of genotype; all others measured phenotype

**Figure 1** Odds ratios (ORs) for bladder cancer in NAT2 slow vs NAT2 fast acetylators in 21 case–control studies and for those not occupationally exposed. The relationship between smoking *per se* and NAT2 status in the study populations was evaluated by calculating the odds ratio for smoking in NAT2 slow compared with NAT2 fast acetylators in the controls from the five studies providing sufficient information. Summary odds ratios were calculated by the Mantel–Haenszel method. The method of empirically weighted least squares was used to test for heterogeneity within and between the odds ratios for each of the groups or subgroups of studies (Cox and Snell, 1989).

The evidence for the associations investigated in this paper has been summarized graphically in Figures 1 to 3. In Figures 1 and 3, studies are listed in order of study size (number of cases), from largest to smallest. In Figure 2, studies are listed in order of date of publication, earliest first, within each group. In these Figures, black squares indicate odds ratios, the area of each square being proportional to the amount of statistical information contributed, with horizontal lines indicating 95% confidence intervals. Vertical dashed lines represent point estimates of summary odds ratios. Confidence intervals for summary odds ratios were calculated but have not been included in the Figures because we believe that they may underestimate substantially the degree of uncertainty in the results due to publication bias and to limitations in the design of studies.

### RESULTS

In total, 27 published studies were identified. Of these, 21 met the eligibility criteria (Lower et al, 1979 (two studies); Cartwright et al, 1982; Evans et al, 1983; Hanssen et al, 1985; Ladero et al, 1985; Mommsen et al, 1985; Sone, 1986; Kaisary et al, 1987; Horai et al, 1989; Roots et al, 1989; Hanke and Krajewska, 1990; Lee et al, 1994; Dewan et al, 1995; Ishizu et al, 1995; Risch et al, 1995; Brockmöller et al, 1996; Okkels et al, 1997; Peluso et al, 1998; Su et al, 1998; Taylor et al, 1998). Of the remaining six studies, five had fewer than 50 cases (Woodhouse et al, 1982; Miller and Cosgriff, 1983; Karakaya et al, 1986; Bicho et al, 1988; Hayes et al, 1993) and one gave insufficient information on methods and results to allow calculation of odds ratios (Probert et al, 1998). These studies were not included in the analysis.

The 21 studies included in the analysis varied considerably in size and in design and most did not provide sufficient information to allow a full assessment of the relationship between the NAT2 polymorphism and smoking or occupational exposure in relation to bladder cancer risk. For example, 2/21 studies gave response rates and 12/21 studies stated whether cases were incident or prevalent. Five studies provided information on smoking for both cases and controls in sufficient detail to allow the calculation of

*© 2000 Cancer Research Campaign*

| Study | Cases/Controls NAT2 slow | Cases/Controls NAT2 fast | OR |
|---|---|---|---|
| **Studies conducted in Japan, Korea and Taiwan** | | | |
| Sone 1986 | 5/11 | 57/71 | 0.57 |
| Horai et al 1989 | 3/13 | 48/190 | 0.91 |
| Lee et al 1994 | 16/14 | 82/70 | 0.98 |
| Ishizu et al 1995 | 20/13 | 51/78 | 2.35 |
| Su et al 1998 | 12/13 | 41/88 | 1.98 |
| Test for heterogeneity between studies: $\chi^2_4 = 5.25$; $p = 0.26$ | | | |
| **Studies conducted in Europe, India and North America** | | | |
| Lower et al 1979–Denmark | 80/79 | 35/39 | 1.13 |
| Lower et al 1979–Sweden | 46/38 | 25/36 | 1.74 |
| Catwright et al 1982 | 74/54 | 37/41 | 1.52 |
| Evans et al 1983 | 66/510 | 34/342 | 1.30 |
| Hanssen et al 1985 | 65/18 | 40/24 | 2.17 |
| Ladero et al 1985 | 83/90 | 47/67 | 1.31 |
| Mommsen et al 1985 | 145/54 | 83/46 | 1.49 |
| Kaisary et al 1987 | 59/54 | 39/56 | 1.57 |
| Roots et al 1989 | 67/149 | 35/143 | 1.84 |
| Hanke et al 1990 | 47/10 | 20/12 | 2.82 |
| Risch et al 1995 | 127/26 | 62/33 | 2.60 |
| Dewan et al 1995 | 46/28 | 31/52 | 2.76 |
| Brockmoller et al 1996 | 233/215 | 141/158 | 1.21 |
| Okkels et al 1997 | 154/135 | 100/107 | 1.22 |
| Peluso et al 1998 | 76/26 | 38/20 | 1.54 |
| Taylor et al 1998 | 121/109 | 109/94 | 0.96 |
| Test for heterogeneity between studies: $\chi^2_{20} = 19.74$; $p = 0.14$ | | | |
| **ALL STUDIES** | 1545/1659 | 1155/1767 | 1.41 |
| Test for heterogeneity between studies: $\chi^2_{20} = 25.90$; $p = 0.17$ | | | |

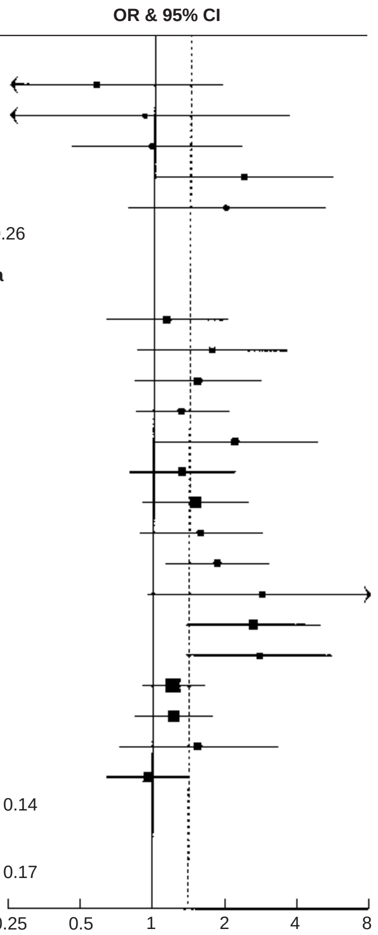

Dashed line represents point estimate of summary odds ratio for all 21 studies

**Figure 2** Odds ratios (ORs) for bladder cancer in NAT2 slow vs NAT2 fast acetylators: by ethnic group odds ratios for bladder cancer risk in NAT2 slow vs fast acetylators stratified by smoking (Dewan et al, 1995; Ishizu et al, 1995; Risch et al, 1995; Brockmöller et al, 1996; Taylor et al, 1998). Smoking was also measured for cases and controls in the study by Okkels et al (1997) but published figures were insufficient to allow stratified analysis comparable with the other studies. In a further five studies smoking data were given only for cases (Cartwright et al, 1982; Hanssen et al, 1985; Horai et al, 1989; Ladero et al, 1985, all of which included both smokers and non-smokers among cases; Evans et al, 1983, in which all cases were smokers). It is not possible to calculate comparable odds ratios of the risk of bladder cancer for NAT2 slow vs fast separately for smokers and non-smokers in these studies. We did not consider that a case-only analysis of the potential interaction between NAT2 and smoking would be justified; this would depend crucially on independence between NAT2 acetylation status and smoking status, which has not been fully established.

Information on occupational exposure to arylamines was given for both cases and controls in four studies (Sone, 1986; Kaisary et al, 1987; Lee et al, 1994; Brockmöller et al, 1996); in two of these, none of the subjects was occupationally exposed (Kaisary et al, 1987; Lee et al, 1994). Six studies included both occupationally exposed and non-occupationally exposed cases, but gave no information on occupational exposure for controls and therefore could not be included in the analyses (Cartwright et al, 1982; Evans et al, 1983; Hanssen et al, 1985; Ladero et al, 1985; Hanke and Krajewska, 1990; Risch et al, 1995). Definitions of smoking and occupational exposure varied widely between studies.

Figure 1 shows the odds ratios for bladder cancer in NAT2 slow acetylators compared with NAT2 fast acetylators in the 21 eligible studies. Estimates ranged from 0.57–2.82, with four studies showing a statistically significant positive association between NAT2 slow and risk of bladder cancer. There was no significant statistical heterogeneity between studies overall ($\chi^2_{20} = 25.90$, $P = 0.16$), and no significant heterogeneity within or between groups of studies based on type of control group (hospital urology, hospital general, population or mixed hospital and population controls; between groups $\chi^2_3 = 2.80$, $P = 0.43$), method used to determine acetylation status (phenotype or genotype; between groups $\chi^2_1 = 2.33$, $P = 0.13$, studies indicated on Figure 1) or ethnic group (East Asian compared with Caucasian; between groups $\chi^2_1 = 0.07$, $P = 0.79$, Figure 2). The point estimate of the summary odds ratio for all 21 studies was 1.41, based on a total of 2700 cases and 3426 controls. The 95% confidence limits for this estimate were 1.26–1.59, but are of questionable value given the limitations outlined in the Discussion.

*© 2000 Cancer Research Campaign*

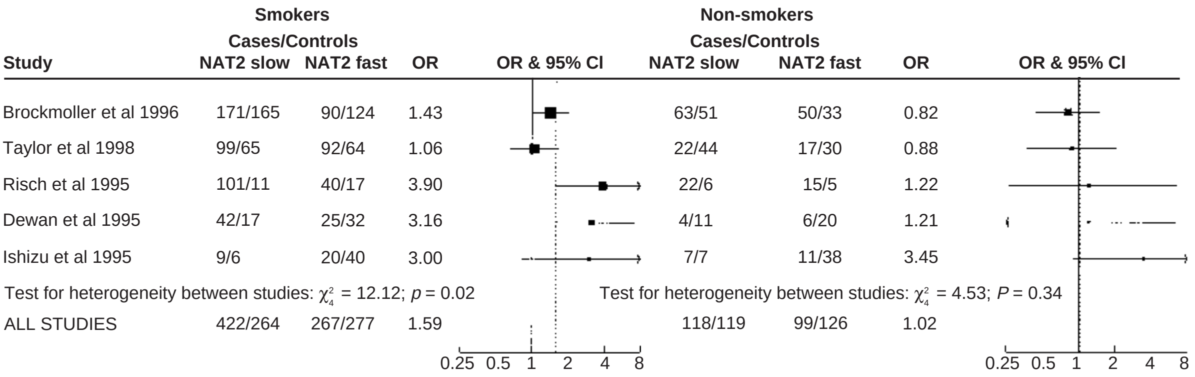

| Study | Smokers Cases/Controls NAT2 slow | NAT2 fast | OR | Non-smokers Cases/Controls NAT2 slow | NAT2 fast | OR |
|---|---|---|---|---|---|---|
| Brockmoller et al 1996 | 171/165 | 90/124 | 1.43 | 63/51 | 50/33 | 0.82 |
| Taylor et al 1998 | 99/65 | 92/64 | 1.06 | 22/44 | 17/30 | 0.88 |
| Risch et al 1995 | 101/11 | 40/17 | 3.90 | 22/6 | 15/5 | 1.22 |
| Dewan et al 1995 | 42/17 | 25/32 | 3.16 | 4/11 | 6/20 | 1.21 |
| Ishizu et al 1995 | 9/6 | 20/40 | 3.00 | 7/7 | 11/38 | 3.45 |
| ALL STUDIES | 422/264 | 267/277 | 1.59 | 118/119 | 99/126 | 1.02 |

Smokers: Test for heterogeneity between studies: $\chi^2_4 = 12.12$; $p = 0.02$

Non-smokers: Test for heterogeneity between studies: $\chi^2_4 = 4.53$; $P = 0.34$

Dashed lines represent point estimates of summary odds ratios for all 5 studies

**Figure 3** Odds ratios (ORs) for bladder cancer in NAT2 slow vs NAT2 fast acetylators in smokers and non-smokers from five studies

### Smoking

### *Smoking and the NAT2 polymorphism in people without bladder cancer*

In no study was there a significant association between the NAT2 polymorphism and smoking status and there was no significant heterogeneity between studies ($p = 0.77$). The summary odds ratio for smoking in NAT2 slow vs NAT2 fast acetylators in controls from five studies was 0.79 (0.56–1.11), based on a total of 541 smokers and 245 non-smokers.

### *Relationship between the NAT2 polymorphism and bladder cancer in smokers compared with non-smokers*

Figure 3 shows the odds ratios for bladder cancer in NAT2 slow vs NAT2 fast acetylators in smokers and in non-smokers in the five studies with this information. In no individual study was there a significant difference at the 5% level between the odds ratio in smokers and that in non-smokers. There was significant heterogeneity between studies in the smoking subgroup ($p = 0.02$). Point estimates of summary odds ratios were 1.59 for smokers (689 cases and 541 controls) and 1.02 for non-smokers (217 cases and 245 controls). In view of the heterogeneity of results in smokers, no statistical comparison of the summary odds ratios for bladder cancer in NAT2 slow vs NAT2 fast acetylators in smokers and non-smokers was attempted.

Interaction between genetic and environmental risk factors may also be evaluated by means of an ‘odds ratio for interaction’ (Hwang et al, 1994) or ‘interaction odds ratio’ (Cuzick, 1999), defined as:

> odds ratio for bladder cancer in NAT2 slow vs fast acetylators in smokers/odds ratio for bladder cancer in NAT2 slow vs fast acetylators in non-smokers and providing a direct comparison between the effects of NAT2 in smokers and in non-smokers within each study. In view of the unreliability of individual study estimates and the possibility of publication bias this calculation was not performed.

### Occupational exposure

### *Occupational exposure and the NAT2 polymorphism in people without bladder cancer*

The relationship between the NAT2 polymorphism and whether or not individuals were occupationally exposed to arylamines was evaluated in controls from the only study providing the necessary information (Brockmöller et al, 1996). The odds ratio for occupational exposure in slow compared with fast acetylators was 0.74 (0.43–1.26), based on 77 occupationally exposed and 296 non-occupationally exposed subjects.

### *Relationship between the NAT2 polymorphism and bladder cancer in occupationally exposed subjects compared with non-occupationally exposed subjects*

Odds ratios for bladder cancer for NAT2 slow vs NAT2 fast acetylators in occupationally exposed and non-occupationally exposed subjects were calculated for the two studies with sufficient information on exposure and outcome. Sone (1986) found odds ratios of 0.52 (0.07–2.96) in occupationally exposed subjects and 1.85 (0.15–48.69) in non-occupationally exposed subjects; for Brockmöller et al (1996) odds ratios were 1.55 (0.85–2.83) in occupationally exposed and 1.16 (0.80–1.67) in non-occupationally exposed subjects. In neither study were the odds ratios for exposed and non-exposed subjects significantly different from each other. These studies differed in their definition of occupational exposure and no combined analysis was attempted.

### DISCUSSION

Interest in the possible role of the NAT2 polymorphism in determining susceptibility to bladder cancer followed identification of industrial arylamines as potent bladder carcinogens (Case et al, 1954), and recognition of the role of *N*-acetyltransferases in arylamine metabolism (Hein, 1988). Involvement of NAT2 in smoking-related bladder cancer was suggested by identification of arylamines in cigarette smoke and has been supported by studies on haemoglobin- and DNA-adducts (Vineis, 1992) and by recent work on somatic mutation patterns in bladder cancers (Sorlie et al, 1998). Early case-control studies reported a strong association between the NAT2 slow phenotype and bladder cancer in subjects occupationally exposed to arylamines, with odds ratios for bladder cancer in NAT2 slow vs fast acetylators as high as 16.7 (Cartwright et al, 1982; Ladero et al, 1985; Hanke and Krajewska, 1990). In all of these early studies occupationally exposed subjects were compared with controls whose occupational exposure status was not known. Subsequent studies with varying extents and types of environmental exposure have produced inconsistent results.

*© 2000 Cancer Research Campaign*

Few studies, however, have explicitly tested the hypothesis of interaction between NAT2 and smoking or occupational exposure in relation to bladder-cancer risk, although this is one of the best theoretical examples available of possible gene–environment interaction. To test for the effects of specific genetic and environmental risk factors, and to examine any possible statistical interaction between them, both types of exposure need to be measured accurately in cases and in controls. Only five of the studies discussed here gave adequate information on smoking for cases and controls, and occupational exposure was measured in cases and controls in only two studies. None of these studies was designed in such a way that the individual effects on bladder-cancer risk of the NAT2 polymorphism and of the environmental exposure could both be measured. The majority of studies thus provide only an overall measure of the effect of NAT2 on bladder-cancer risk, and the lack of information about environmental exposure makes these estimates difficult to compare.

Interpretation of the results of the 21 case-control studies reviewed here is limited also by the unreliability of individual study results and by possible selective publication of positive results (publication bias). The majority of studies gave inadequate information on case and control selection. Case definition was generally poor and in many studies no histological confirmation of diagnosis was required; sub-types of cancer, including the two common types of superficial transitional cell carcinoma of the bladder, may vary in their genetic and environmental aetiologies (Knowles, 1998; Le Marchand et al, 1998; Whiteman et al, 1998). The selective publication of studies (in particular small studies) with positive results is common and means that the validity of any summary measure is open to question (Egger et al, 1998). There is some evidence to suggest such publication bias among the studies reviewed here (Figures 1 and 3), with the odds ratios for bladder cancer in NAT2 slow vs NAT2 fast acetylators being higher in the smaller studies, especially among smokers.

Bearing in mind these difficulties in interpretation, it is possible to draw some useful conclusions from the data reviewed here. First, compared with the substantial risks of bladder cancer associated with smoking and occupational exposure to arylamines, the effect of acetylation status on an individual's risk of developing bladder cancer, if there is any effect at all, is likely to be small. The overall summary risk estimate suggests an increase in risk of about 40% associated with the NAT2 slow phenotype or genotype. Any gene–environment interaction also seems likely to be small; although the summary odds ratio for bladder cancer associated with NAT2 slow was estimated at 1.6 in smokers and 1.0 in non-smokers, it should be noted that this difference may have been exaggerated by publication bias. Secondly, we found no significant difference in the effect of NAT2 on bladder-cancer risk between studies in Caucasian (European, Indian and North American) and those in East Asian (Chinese, Japanese and Korean) populations, or between studies based on phenotype and studies based on genotype. In two previous meta-analyses, d'Errico et al (1996; 1999) found an association between NAT2 slow and bladder-cancer risk only in Caucasians. They, however, had fewer East Asian studies available, and indeed in the present analysis the few East Asian studies were too small to allow an accurate estimation of the effect of NAT2 slow in East Asian populations. Agreement between phenotype and genotype studies is not surprising; NAT2 is not inducible by its substrates, and the NAT2 polymorphism is very well characterized with excellent genotype–phenotype correlation (Smith et al, 1997; Hirvonen, 1999). At least nine different mutated ('slow') alleles, resulting from combinations of at least seven different base-pair substitutions (mutations) have been identified for NAT2 (Vatsis et al, 1995; Hirvonen, 1999), and different slow alleles may be associated with differences in acetylation rate (Cascorbi et al, 1995).

If the effect of NAT2 on bladder-cancer risk is of the size suggested by these summary figures, full examination of the effect within particular exposure groups, for example smokers and non-smokers, will require studies with very large numbers. At least 600 cases and 600 controls would be needed to detect an overall odds ratio (ignoring the effect of environmental exposure) for the effect of NAT2 slow vs NAT2 fast of 1.4 with 80% power and 5% significance, nearly twice the size of the largest available published study. In order to detect a difference of 1.6-fold in the effect on bladder cancer risk of NAT2 slow vs NAT2 fast in smokers compared with non-smokers (assuming no effect of NAT2 in non-smokers, an odds ratio of 3 for the effect of smoking on bladder-cancer risk, a prevalence of NAT2 slow acetylator status in controls of 0.5 and prevalence of smoking in cases and controls of 0.5), an unmatched case-control study would require at least 3000 cases and 3000 controls. The sample size is very sensitive to small changes in parameter; for example, to detect a difference in the effect of NAT2 between smokers and non-smokers of 1.5-fold (rather than 1.6-fold) would require at least 5000 cases and 5000 controls. The required study size would need to be further increased to allow for adjustment for confounding, for the effects of misclassification of gene or exposure status (Rothman et al, 1999), for modelling the results by levels of exposure and/or for possible interactions with other gene polymorphisms (e.g. *N*-acetyltransferase 1 (NAT1); Hirvonen, 1999) or with other environmental exposures. Although desirable, studies dealing with several different risk factors and possible interactions between them need to be interpreted cautiously in order to avoid the danger of over-emphasizing statistically significant results in small subgroups (Oxman and Guyatt, 1992; Cuzick, 1999).

### ACKNOWLEDGEMENTS

We thank D. R. Cox and Gillian Reeves for statistical advice, Paul Appleby for help with Figures, and Valerie Beral, Tim Bishop, Margaret Knowles and Peter Selby for helpful comments.

### REFERENCES

Bicho MP, Breitenfeld L, Carvalho AA and Manso CF (1988) Acetylation phenotypes in patients with bladder carcinoma. *Ann Genet* **31**: 167–171

Brockmöller J, Cascorbi I, Kerb R and Roots I (1996) Combined analysis of inherited polymorphisms in arylamine N-acetyltransferase 2, glutathione S-transferases M1 and T1, microsomal epoxide hydrolase, and cytochrome P450 enzymes as modulators of bladder cancer risk. *Cancer Res* **56**: 3915–3925

Cartwright RA (1979) Genetic association with bladder cancer. *BMJ* **2**: 798

Cartwright RA, Glashan RW, Rogers HJ, Ahmad RA, Barham-Hall D, Higgins E and Kahn MA (1982) Role of N-acetyltransferase phenotypes in bladder carcinogenesis: a pharmacogenetic epidemiological approach to bladder cancer. *Lancet* **2**: 842–845

Cascorbi I, Drakoulis N, Brockmöller J, Maurer A, Sperling K and Roots I (1995) Arylamine N-acetyltransferase (*NAT2*) mutations and their allelic linkage in unrelated caucasian individuals: correlation with phenotypic activity. *Am J Hum Genet* **57**: 581–592

Case RA, Hosker ME, McDonald DB and Pearson JT (1954) Tumours of the urinary bladder in workmen engaged in the manufacture and use of certain dyestuff intermediates in the British chemical industry. *British Journal of Industrial Medicine* **11**: 75–104

 *© 2000 Cancer Research Campaign*

Cox DR and Snell EJ (1989) *Analysis of Binary Data*, p. 60. Chapman and Hall: London

Cuzick J (1999) Interaction, subgroup analysis and sample size. In: *Metabolic Polymorphisms and Susceptibility to Cancer*, Vineis P, Malats N, Lang M, d'Errico A, Caporaso N, Cuzick J and Boffetta P (eds), pp. 109–121. International Agency for Research on Cancer: Lyon d'Errico A, Taioli E, Chen X and Vineis P (1996) Genetic metabolic polymorphisms and the risk of cancer: a review of the literature. *Biomarkers* **1**: 149–173

d'Errico A, Malats N, Vineis P and Boffetta P (1999) Review of studies of selected metabolic polymorphisms and cancer. In: *Metabolic Polymorphisms and Susceptibility to Cancer*, Vineis P, Malats N, Lang M, d'Errico A, Caporaso N, Cuzick J and Boffetta P (eds), pp. 323–393. International Agency for Research on Cancer: Lyon

Dewan A, Chattopadhyay P and Kulkarni PK (1995) N-acetyltransferase activity – a susceptibility factor in human bladder carcinogenesis. *Indian J Cancer* **32**: 15–19

Egger M, Schneider M and Davey Smith G (1998) Meta-analysis: Spurious precision? Meta-analysis of observational studies. *BMJ* **316**: 140–144

Evans DAP, Eze LC and Whibley EJ (1983) The association of the slow acetylator phenotype with bladder cancer. *J Med Genet* **20**: 330–333

Grant DM, Hughes NC, Janezic SA, Goodfellow GH, Chen HJ, Gaedigk A, Yu VL and Grewal R (1997) Human acetyltransferase polymorphisms. *Mutat Res* **376**: 61–70

Hanke J and Krajewska B (1990) Acetylation phenotypes and bladder cancer. *Journal of Occupational Medicine* **32**: 917–918

Hanssen HP, Agarwal DP, Goedde HW, Bucher H, Huland H, Brachmann W and Ovenbeck R (1985) Association of N-acetyltransferase polymorphism and environmental factors with bladder carcinogenesis. Study in a north German population. *Eur Urol* **11**: 263–266

Hayes RB, Bi W, Rothman N, Broly F, Caporaso N, Feng P, You X, Yin S, Woosley RL and Meyer UA (1993) N-Acetylation phenotype and risk of bladder cancer in benzidine-exposed workers. *Carcinogenesis* **14**: 675–678

Hein DW (1998) Acetylator phenotype and arylamine-induced carcinogenesis. *Biochim Biophys Acta* **948**: 37–66

Hirvonen A (1999) Polymorphic NATs and cancer predisposition. In: *Metabolic Polymorphisms and Susceptibility to Cancer*, Vineis P, Malats N, Lang M, d'Errico A, Caporaso N, Cuzick J and Boffetta P (eds), pp. 251–270. International Agency for Research on Cancer: Lyon

Horai Y, Fujita K and Ishizaki T (1989) Genetically determined N-acetylation and oxidation capacities in Japanese patients with non-occupational urinary bladder cancer. *Eur J Clin Pharmacol* **37**: 581–587

Hwang S-J, Beaty TH, Liang K-L, Coresh J and Khoury MJ (1994) Minimum sample size estimation to detect gene-environment interaction in case-control designs. *Am J Epidemiol*, **140**: 1029–1037

Ishizu S, Hashida C, Hanaoka T, Maeda K and Ohishi Y (1995) N-acetyltransferase activity in the urine in Japanese subjects: comparison in healthy persons and bladder cancer patients. *Jpn J Cancer Res* **86**: 1179–1181

Kaisary A, Smith P, Jaczq E, McAllister CB, Wilkinson GR, Ray WA and Branch RA (1987) Genetic predisposition to bladder cancer: ability to hydroxylate debrisoquine and mephenytoin as risk factors. *Cancer Res* **47**: 5488–5493

Kalo R, Estabrook RW and Cayen MN (eds) pp 449–506. Taylor and Francis: London

Kantor AF, Hartge P, Hoover RN and Fraumeni JF Jr (1985) Familial and environmental interactions in bladder cancer risk. *Int J Cancer* **35**: 703–706

Karakaya AE, Cok I, Sardas S, Gogus O and Sardas OS (1986) N-Acetyltransferase phenotype of patients with bladder cancer. *Human Toxicology* **5**: 333–335

Kiemeny LA, Moret NC, Witjes JA, Schoenberg MP and Tulinius H (1997) Familial transitional cell carcinoma among the population of Iceland. *J Urol* **157**: 1649–1651

Knowles MA (1998) Molecular genetics of bladder cancer: pathways of development and progression. In: *Cancer Surveys 31: Bladder Cancer*, Oliver RTD, Coptcoat MJ (eds), pp. 49–76. Imperial Cancer Research Fund-Cold Spring Harbour Laboratory Press: New York

Ladero JM, Kwok CK, Jara C, Fernandez L, Silmi AM, Tapia D and Uson AC (1985) Hepatic acetylator phenotype in bladder cancer patients. *Annals of Clinical Research* **17**: 96–99

Lee SW, Jang IJ, Shin SG, Lee KH, Yim DS, Kim SW, Oh SJ and Lee SH (1994) CYP1A2 activity as a risk factor for bladder cancer. *J Korean Med Sci* **9**: 482–489

Le Marchand LL, Sivaraman L, Pierce L, Seifried A, Lum A, Wilkens A and Lau AF (1998) Associations of *CYP1A1*, *GSTM1*, and *CYP2E1* polymorphisms with lung cancer suggest cell type specificities to tobacco carcinogens. *Cancer Res* **58**: 4858–4863

Lin HJ, Han C-Y, Lin BK and Hardy S (1994) Ethnic distribution of slow acetylator mutations in the polymorphic N-acetyltransferase (NAT2) gene. *Pharmacogenetics* **4**: 125–134

Lower GM, Jr., Nilsson T, Nelson CE, Wolf H, Gamsky TE and Bryan GT (1979) N-acetyltransferase phenotype and risk in urinary bladder cancer: approaches in molecular epidemiology. Preliminary results in Sweden and Denmark. *Environ Health Perspect* **29**: 71–79

Miller ME and Cosgriff JM (1983) Acetylator phenotype in human bladder cancer. *J Urol* **130**: 65–66

Mommsen S, Barfod NM and Aagaard J (1985) N-Acetyltransferase phenotypes in the urinary bladder carcinogenesis of a low-risk population. *Carcinogenesis* **6**: 199–201

Okkels H, Sigsgaard T, Wolf H and Autrup H (1997) Arylamine N-acetyltransferase 1 (NAT1) and 2 (NAT2) polymorphisms in susceptibility to bladder cancer: the influence of smoking. *Cancer Epidemiol Biomarkers Prev* **6**: 225–231

Oxman AD and Guyatt GH (1992) A consumer's guide to subgroup analysis. *Ann Intern Med* **116**: 79–84

Peluso M, Airoldi L, Armelle M, Martone T, Coda R, Malaveille C, Giacomelli G, Terrone C, Casetta G and Vineis P (1998) White blood cell DNA adducts, smoking, and *NAT2* and *GSTM1* genotypes in bladder cancer: a case-control study. *Cancer Epidemiol Biomarkers Prev* **7**: 341–346

Probert JL, Persad RA, Greenwood RP, Gillatt DA and Smith PJB (1998) Epidemiology of transitional cell carcinoma of the bladder: profile of an urban population in the south-west of England. *Br J Urol* **82**: 660–666

Risch A, Wallace DM, Bathers S and Sim E (1995) Slow N-acetylation genotype is a susceptibility factor in occupational and smoking related bladder cancer. *Hum Mol Genet* **4**: 231–236

Roots I, Drakoulis N, Brockmöller J, Janicke I, Cuprunov M and Ritter J (1989) Hydroxylation and acetylation phenotypes as genetic risk factors in certain malignancies. In: Kato R, Estabrook RW and Cayen MN (eds) *Xenobiotic Metabolism and Disposition.* pp 499–506. Taylor and Francis: London

Rothman N, Garcia-Closas M, Stewart WT and Lubin J (1999) The impact of misclassification in case-control studies of gene-environment interactions. In: *Metabolic Polymorphisms and Susceptibility to Cancer*, Vineis P, Malats N, Lang M, d'Errico A, Caporaso N, Cuzick J and Boffetta P (eds), pp. 89–96. International Agency for Research on Cancer: Lyon

Silverman DT, Morrison AS and Devesa SS (1996) Bladder cancer. In: *Cancer Epidemiology and Prevention*, Schottenfeld D, Fraumeni JF (eds), pp. 1156–1179. OUP: New York, Oxford

Smith CAD, Wadelius M, Gough AC, Harrison DJ, Wolf CR and Rane A (1997) A simplified assay for the arylamine N-acetyltransferase 2 polymorphism validated by phenotyping with isoniazid. *J Med Genet* **34**: 758–760

Sone M (1986) [Determination of the N-acetyltransferase phenotype in urothelial cancer patients and healthy controls]. [Japanese]. *Hinyokika Kiyo* **32**: 1085–1092

Sorlie T, Martel-Planche G, Hainaut P, Lewalter J, Holm R, Borresen-Dale A-L and Montesano R (1998) Analysis of *p53*, $p16^{MTS1}$. $p21^{WAF1}$ and *H-ras* in archived bladder tumours from workers exposed to aromatic amines. *Br J Cancer* **77**: 1573–1579

Su HJ, Guo YL, Lai M-D, Huang J-D, Cheng Y and Christiani DC (1998) The NAT2* slow genotype is associated with bladder cancer in Taiwanese, but not in the Black Foot Disease endemic area population. *Pharmacogenetics* **8**: 187–190

Taylor JA, Umbach DM, Stephens E, Castranio T, Paulson D, Robertson C, Mohler JL and Bell DA (1998) The role of *N*-acetylation polymorphisms in smoking-associated bladder cancer: evidence of a gene-gene-exposure three-way interaction. *Cancer Res* **58**: 3603–3610

Vatsis KP, Weber WW, Bell DA, Dupret J-M, Price Evans DA, Grant DM, Hein DW, Lin HJ, Meyer UA, Relling MV, Sim E, Suzuki T and Yamazoe Y (1995) Nomenclature for N-acetyltransferases. *Pharmacogenetics* **5**: 1–17

Vineis P (1992) Epidemiological models of carcinogenesis: the example of bladder cancer. *Cancer Epidemiol Biomarkers Prev* **1**: 149–153

Vineis P, Malats N, Lang M, d'Errico A, Caporaso N and Boffetta P (eds) (1999) *Metabolic Polymorphisms and Susceptibility to Cancer*. International Agency for Research on Cancer: Lyon

Whiteman DC, Parsons PG and Green A (1998) p53 expression and risk factors for cutaneous melanoma: a case-control study. *Int J Cancer* **77**: 843–848

Woodhouse KW, Adams P, Clothier A, Mucklow J and Rawlins MD (1982) N-acetylation phenotype in bladder cancer. *Human Toxicology* **1**: 443–445

Woolhouse NM, Qureshi MM, Bastaki SMA, Patel M, Abdulrazzaq Y and Bayoumi RAL (1997) Polymorphic N-acetyltransferase (NAT2) genotyping of Emiratis. *Pharmacogenetics* **7**: 73–82

Xie H-G, Xu Z-H, Ou-Yang D-S, Shu Y, Yang D-L, Wang J-S, Yan Z-D, Huang S-L, Wang W and Zhou H-H (1997) Meta-analysis of phenotype and genotype of NAT2 deficiency in Chinese populations. *Pharmacogenetics* **7**: 503–514

© 2000 Cancer Research Campaign
